# Complex Thoracic Resections in the Minimally Invasive Era: Is Open Surgery Becoming a Lost Skill?

**DOI:** 10.3390/jcm15135135

**Published:** 2026-07-01

**Authors:** Giacomo Argento, Erino Angelo Rendina, Giulio Maurizi

**Affiliations:** Department of Thoracic Surgery, Sapienza University of Rome Sant’Andrea Hospital, 00189 Rome, Italy; erinoangelo.rendina@uniroma1.it (E.A.R.); giulio.maurizi@uniroma1.it (G.M.)

**Keywords:** thoracic surgery, minimally invasive surgery, VATS, open thoracotomy, surgical training, sleeve lobectomy

## Abstract

The rapid expansion of video-assisted thoracoscopic surgery (VATS) and robotic-assisted thoracic surgery (RATS) has reshaped thoracic surgical practice over the last two decades, offering reduced perioperative morbidity, shorter hospital stay, and oncological outcomes comparable to conventional thoracotomy in appropriately selected patients. Minimally invasive techniques now account for the majority of anatomical pulmonary resections in many high-volume centers and are being explored, in selected patients at experienced institutions, for increasingly complex procedures. This shift, however, raises a question that has received comparatively little attention: whether reduced trainee exposure to open thoracotomy may, over time, erode open thoracic surgical competence. As minimally invasive approaches become the institutional default, exposure to open surgery is declining, and the skills required to perform complex open resections or to manage intraoperative emergencies may become confined to a diminishing cohort of senior surgeons. In this narrative review, we examine the current boundaries of minimally invasive thoracic surgery, define the clinical scenarios in which open surgery remains indispensable—including bronchoplastic and angioplastic resections, post-induction hostile surgical fields, and unplanned conversion—and consider the implications of the ongoing paradigm shift for training, taking into account the substantial variability of thoracic surgical practice across different regions. We argue that open thoracic surgery is not an obsolete discipline but a foundational competence whose preservation may warrant deliberate attention through structured exposure, simulation, mentorship, and dedicated competence assessment. Throughout, we have sought to distinguish documented trends from reasonable concern and from speculative future risk, and we frame the central issue explicitly as a credible and foreseeable risk rather than a demonstrated decline.

## 1. Introduction

The history of thoracic surgery is, in many respects, a history of progressive restraint, of attempting to achieve the same oncological objective with less access trauma. For most of the twentieth century, the posterolateral thoracotomy represented the default route for major pulmonary and mediastinal resection, and the conceptual and technical foundations of the specialty—anatomical lobectomy, systematic nodal dissection, bronchoplastic and angioplastic reconstruction, and the management of the great vessels of the chest—were established within the open field. From this foundation, the field has undergone a profound and accelerating transformation, driven by the advent and maturation of minimally invasive techniques.

**Figure 1:**
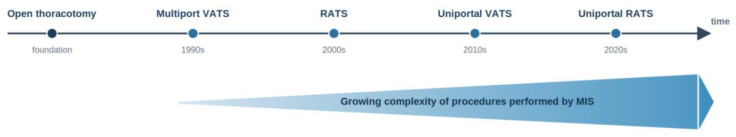
Evolution of access in thoracic surgery, from open thoracotomy to multiport VATS, uniportal VATS, robotic-assisted thoracic surgery (RATS), and the current exploration of complex minimally invasive resection. The progression illustrates the directional reduction in access trauma over time; it is not intended to imply that more recent approaches have replaced open surgery across all indications.

Video-assisted thoracoscopic surgery (VATS), introduced into clinical practice in the early 1990s, was initially met with skepticism by a surgical community accustomed to the exposure and tactile feedback of the open field. Over subsequent decades, however, randomized and comparative data have consistently shown advantages in postoperative pain, quality of life, length of hospital stay, and perioperative recovery, while supporting oncological outcomes comparable to open lobectomy in appropriately selected patients [1,2]. The trajectory did not stop at lobectomy. The progressive refinement of instrumentation, optics, and surgical technique, including the development of uniportal VATS, extended the reach of thoracoscopy into increasingly demanding territory: segmentectomy, sleeve resections, and more recently procedures once considered the exclusive domain of open surgery [3,4,5,6]. The parallel emergence of robotic-assisted thoracic surgery (RATS) has further expanded this frontier, offering enhanced three-dimensional visualization, articulated instrument movement, and improved ergonomics that partially overcome the inherent constraints of rigid thoracoscopic tools [7,8]. The arc of this evolution, from open thoracotomy, through multiport and then uniportal VATS, to RATS and the current exploration of complex minimally invasive resection, is summarized in Figure 1.

This evolution carries a consequence that has received comparatively little attention: the possibility of a gradual erosion of open surgical competence. As VATS and RATS become the default approach in many training programs and high-volume institutions, the exposure of thoracic surgery trainees to open thoracotomy is declining, in some settings at a pace that outstrips any deliberate curricular planning. The contemporary thoracic surgical trainee may therefore inhabit a different operative environment from that of a fellow a generation earlier. The potential implications touch on patient safety during unplanned conversion, the capacity of institutions to manage complex thoracic emergencies, and the long-term sustainability of open surgical expertise as a discipline.

This narrative review examines the current state of minimally invasive thoracic surgery, delineates the clinical scenarios in which open surgery remains indispensable, situates these issues within the considerable international variability of thoracic surgical practice, and interrogates what the ongoing paradigm shift may mean for the training and competence assessment of the next generation of thoracic surgeons. Our central contention is not that open surgery should resist innovation; rather, we argue that the preservation of open surgical competence should be regarded as a distinct responsibility of the thoracic surgical community, to be addressed deliberately rather than left to informal apprenticeship alone.

## 2. Literature Search

This is a narrative review and was not conducted as a systematic review; no formal meta-analytic synthesis or risk-of-bias assessment was performed. Nonetheless, to improve transparency and to reduce the risk of selective citation, we describe here how the literature was identified and selected. We searched PubMed/MEDLINE and Scopus for English-language articles published between January 2000 and January 2026, combining the terms “video-assisted thoracoscopic surgery”, “VATS”, “robotic-assisted thoracic surgery”, “RATS”, “thoracotomy”, “sleeve lobectomy”, “carinal resection”, “superior sulcus tumor”, “conversion”, “surgical training”, “learning curve”, “simulation”, and “competence assessment”, using Boolean operators. Reference lists of relevant articles were screened manually for additional sources, and key society documents on thoracic surgical training were consulted.

Given the narrative and argumentative nature of the review, article selection was purposive rather than exhaustive. We prioritized randomized trials, meta-analyses, large national database analyses, and multicenter series where available, and supplemented these with high-quality single-center reports, technical descriptions, and expert consensus documents when comparative data were lacking—most notably for complex minimally invasive procedures, where the literature consists predominantly of small series from a limited number of institutions. Where the evidence base is thin or conflicting, we have tried to indicate this explicitly rather than to present individual reports as representative of general practice.

## 3. The Persistent Domain of Open Surgery

Before examining the implications of the minimally invasive surgery (MIS) revolution, it is necessary to define the procedures that continue to require, or substantially benefit from, an open approach, and to acknowledge that this domain is neither marginal nor disappearing. These scenarios are summarized in Table 1 and discussed below.

Bronchoplastic and angioplastic resections, most notably sleeve lobectomy, represent the paradigmatic complex procedure in thoracic oncology. Sleeve lobectomy requires precise bronchial transection, tension-free anastomotic reconstruction, and meticulous management of the bronchial vascular supply, and the evidence base consistently favors it over pneumonectomy in suitable patients on both functional and oncological grounds [15]. At the extreme of angioplastic complexity, en bloc resection of locally advanced tumors invading the great vessels, including the thoracic aorta, can be accomplished with acceptable morbidity and mortality in highly selected patients at experienced centers, but mandates open access and advanced vascular reconstruction techniques [9].

Carinal resections, involving resection and reconstruction of the tracheobronchial bifurcation, occupy the apex of technical complexity. These procedures demand mastery of airway management under shared surgical–anesthetic access and command of multiple anastomotic configurations [10,11]. Long-term outcomes from dedicated high-volume centers report R0 resection rates approaching 97–98%, with 30-day mortality around 7% and five-year overall survival of approximately 50% for non-small cell lung cancer [10]—figures that underscore both the oncological validity and the inherent risk of carinal surgery, and that have been achieved only through rigorous patient selection and concentration of these cases in specialized centers.

Superior sulcus tumors require close coordination between thoracic, vascular, and neurosurgical teams. Trimodality treatment, comprising induction chemoradiotherapy followed by surgical resection, has been established as the approach offering the greatest survival benefit, with two-year survival rates of 70–93% reported for the trimodal group versus 22–49% for radiotherapy and surgery alone [12]. Resection therefore invariably occurs in a previously irradiated field, with attendant tissue fragility, altered tissue planes, and an increased risk of complications that substantially amplify operative complexity.

Redo thoracic surgery after prior pulmonary resection or pleurodesis generates hostile pleural fields, with intrapleural adhesions present in up to 76% of reoperations and conversion to open thoracotomy occurring in approximately 12% of initially thoracoscopic redo cases [13]. Patients undergoing resection after neoadjuvant chemoimmunotherapy represent a growing population in whom a minimally invasive initial approach is feasible in approximately half of cases, but in whom the conversion rate reaches 19%, substantially higher than in the upfront setting, reflecting the fibrosis and altered hilar anatomy that systemic therapy may induce [14].

Finally, patients with limited tolerance to single-lung ventilation, severe pleural adhesions, or thoracic deformity may require open thoracotomy as their only viable surgical option; and in emergency thoracic surgery (traumatic injuries, hemoptysis from major vessels, intraoperative catastrophes) open surgical competence must be available at the presenting institution regardless of its minimally invasive profile. Taken together, these scenarios do not represent a residual historical niche, but a clinically relevant domain in which exposure, tactile feedback, rapid vascular control, and reconstructive versatility may remain decisive for patient safety.

## 4. The MIS Revolution: Achievements and Current Boundaries

The adoption of minimally invasive approaches for pulmonary resection has been one of the most rapid transformations in modern thoracic surgery. Analysis of the National Cancer Database demonstrates that, for stage I and II non-small cell lung cancer (NSCLC), the use of minimally invasive lobectomy increased more than fourfold between 2010 and 2017, with the minimally invasive approach surpassing open thoracotomy as the predominant approach for stage I disease as early as 2015, and accounting for more than half of all lobectomies for both stages by 2017 [16]. Conversion rates fell concurrently, from nearly 20% in 2010 to below 8% by 2017 for stage I disease [16], reflecting the maturation of institutional expertise. Significant inter-institutional variability persists, however: adjusted VATS lobectomy rates across US hospitals range from 0.6% in the lowest quintile to 76% in the highest, a disparity that cannot be explained by patient or tumor characteristics alone [17].

The perioperative advantages of VATS over open thoracotomy for anatomical lung resection are well established. A meta-analysis pooling nine studies and over 3700 patients found that VATS lobectomy after induction therapy was associated with significantly shorter operative time, shorter hospital stay, fewer perioperative complications, and less blood loss compared with open lobectomy, with no significant difference in three-year overall or disease-free survival [18]. RATS has further advanced this frontier: in the multicenter PORTaL (Pulmonary Open, Robotic, and Thoracoscopic Lobectomy) consortium, robotic lobectomy was associated with a significantly lower conversion rate compared with VATS—3.6% versus 12.9%—with anatomical difficulty as the predominant reason for VATS conversions [19].

The progressive extension of minimally invasive techniques to complex resections represents the current frontier, and here the evidence base requires careful qualification. Minimally invasive sleeve resection has been shown to be feasible in experienced centers, with series reporting acceptable morbidity and perioperative outcomes comparable to the open approach [20]. Uniportal VATS carinal and tracheobronchial reconstructions have been described in technical detail by dedicated groups, with feasibility documented in selected patients at ultra-high-volume centers [4,5]. For double-sleeve bronchovascular resections, the adoption rate of minimally invasive surgery remains very low even among experienced VATS surgeons [5]. These reports, however, originate almost exclusively from a small number of institutions with exceptional individual expertise, and the absence of multicenter comparative data means that such procedures cannot yet be considered validated minimally invasive alternatives for the general thoracic surgical community. For chest wall resections, superior sulcus tumors, and carinal procedures, the evidence remains confined to small series and hybrid approaches [6], without the comparative data necessary to draw definitive conclusions about equivalence with open techniques. These procedures should therefore continue to be regarded as feasible in expert hands rather than as established alternatives.

## 5. A Global Perspective on Practice Variation

The discussion so far reflects predominantly the experience of high-income, high-volume centers, and any consideration of open surgical competence must take into account the substantial variability of thoracic surgical practice across regions. The adoption of minimally invasive surgery is far from uniform worldwide, and the determinants of this variability are economic and systemic as much as clinical. An analysis of the European Society of Thoracic Surgeons (ESTS) database, comprising 148,628 lung operations performed between 2001 and 2023, found that thoracotomy rates declined significantly over the study period while VATS and RATS adoption increased, with marked country-level variability—faster uptake in countries such as France and Italy and slower adoption in middle-income settings—and a non-linear relationship between national wealth and VATS adoption, whereas no such correlation was observed for RATS [21]. The authors concluded that addressing disparities in access to minimally invasive thoracic surgery requires coordinated efforts to strengthen infrastructure, training, and reimbursement and procurement frameworks [21].

In many low- and middle-income countries, open thoracotomy remains the predominant approach, reflecting the high cost of disposable instrumentation, staplers and energy devices, limited access to advanced platforms, constrained institutional volume, and a shortage of structured minimally invasive training pathways [22]. This has two implications relevant to the present argument. First, the concern that open competence may erode is geographically uneven: in much of the world, open surgery is not at risk of being lost but remains the daily reality of practice, and the more pressing equity problem is the limited availability of minimally invasive options. Second, and conversely, the international mobility of surgeons and the global diffusion of training standards mean that surgeons trained predominantly in minimally invasive environments may practice in, or be called upon to support, settings where open surgery is essential. A balanced view therefore frames open competence not as an issue confined to high-volume Western centers, but as a globally relevant skill whose distribution is uneven, and whose preservation in the high-MIS setting parallels the challenge of expanding minimally invasive access in the low-MIS setting.

## 6. Conversion: A Planned Safety Strategy

Intraoperative conversion from a minimally invasive to an open approach is not a complication and should not be regarded as a failure. It is a surgical decision that reflects either an anticipated limitation or an unanticipated event requiring exposure and control that thoracoscopic access cannot provide, and it is most appropriately understood as a planned safety strategy. Non-hemorrhagic conversions, driven by dense adhesions, anatomical anomalies, or oncological considerations, allow a controlled transition with time to plan the incision. Hemorrhagic conversions demand immediate, confident open hemostatic control.

In the PORTaL cohort, the overall VATS conversion rate was 12.9%, with tumor size and prior neoadjuvant therapy as the strongest independent risk factors [19]. Pulmonary artery injury—from inadvertent instrument contact, stapler–tissue interaction, or traction on a fragile hilar vessel—is the most feared cause of hemorrhagic conversion [23], and its consequences are determined in large part by the speed and quality of the open surgical response [24]. For minimally invasive pneumonectomy, conversion rates remain high at approximately 32% even at experienced centers [25].

The perioperative outcomes of converted cases are broadly comparable to planned open surgery when managed promptly and competently [26]. This reassuring finding, however, derives almost entirely from institutions with significant open surgical expertise—precisely the environment in which a surgeon who converts a VATS case is also capable of performing a competent open thoracotomy. The published conversion-outcome data implicitly assume what they cannot guarantee in a changing training environment: a surgical team capable of managing an open thoracic field confidently and without preparation time. This is an important caveat to consider when looking at favorable conversion series.

Because the safety of conversion depends on a response that cannot be improvised, it is reasonable to argue that institutions should treat emergency conversion as an event to be anticipated and rehearsed rather than merely tolerated. Published algorithms for VATS intraoperative crisis management consistently emphasize that the decision to proceed with, or convert from, a minimally invasive approach must be calibrated against the surgeon’s genuine open surgical experience [24]. This is an explicit acknowledgement, within the minimally invasive literature itself, that open competence is not optional for the thoracoscopic surgeon. In our view, this supports the development of institutional protocols for emergency conversion, including a predefined plan for incision and rapid hilar control, immediate availability of an open thoracotomy and vascular instrument set, and periodic team rehearsal of the conversion sequence, in the same spirit as other surgical crisis management. Such measures are inexpensive relative to their potential benefit and do not depend on the institution’s minimally invasive caseload.

This has direct implications for how surgical competence is acquired and recognized. In many healthcare systems, competence in minimally invasive thoracic surgery is developed through supervised operative exposure, progressive autonomy, local institutional practice, and professional judgment, with limited standardized procedure-specific assessment. While this apprenticeship-based model has historically underpinned surgical training, it may become less reliable when exposure to open thoracotomy, vascular control, and emergency conversion is progressively reduced. Accumulated experience in VATS or RATS may not, therefore, be accompanied by equivalent preparedness to manage the open surgical field when vascular injury, dense hilar fibrosis, or complex oncological anatomy require conversion to thoracotomy.

## 7. Multidisciplinary and Perioperative Preparedness

Several of the scenarios discussed above are as much anesthetic and perioperative challenges as surgical ones, and competence in complex open thoracic surgery cannot be separated from the preparedness of the wider operative team. Carinal and tracheal resection are the clearest example: they require management of a shared airway through techniques ranging from cross-field intubation and jet ventilation to spontaneous-ventilation and extracorporeal strategies in selected cases, choices that cannot be improvised and that depend on interdisciplinary planning tailored to the location of the lesion, the patient’s comorbidities, and the planned reconstruction [27].

The same logic applies to the management of intraoperative crises during minimally invasive surgery. Intolerance of single-lung ventilation, major hemorrhage from a hilar vessel, hemodynamic instability, and the transition to an open field during emergency conversion all require close, rehearsed coordination between the surgical and anesthesia teams, encompassing reliable lung isolation, a plan for rapid restoration of two-lung ventilation, anticipation of massive transfusion, and clear communication during the conversion itself. In our view, crisis planning for these events should be explicitly multidisciplinary and should be rehearsed at the institutional level, since the competence that determines the outcome of an open conversion is not the surgeon’s alone. Framing preparedness as a team property, rather than an individual one, is consistent with the way these complex procedures are actually delivered in experienced centers.

## 8. Training in a Shifting Landscape

The contemporary thoracic surgical trainee may inhabit an operative environment different from that of a fellow a decade ago. National database data from the Society of Thoracic Surgeons (STS) are consistent with this trajectory: the use of open thoracotomy for segmentectomy has declined at a rate of approximately 2.4% per year since 2012, with minimally invasive approaches now accounting for the large majority of anatomical sublobar resections [28]. This shift has occurred without commensurate curricular adaptation in many programs. Even where formal harmonization has been pursued, its scope remains limited in this respect: the ESTS–ERS task-force syllabus was developed to reduce the considerable variation in length, content, and operative experience across European national programs, yet it standardizes the description of the competence to be acquired without prescribing a minimum open operative experience, so that the balance between minimally invasive and open exposure remains largely at the discretion of individual programs [29]. It is important to be precise about what this evidence does and does not show: a documented decline in open operative exposure is an established trend, whereas a resulting decline in open competence is a reasonable concern that the available data do not directly demonstrate.

Some early indicators of this educational challenge have been reported. Survey data from senior and graduating general surgery residents in the United States showed that no program characteristic, including the presence of a dedicated thoracic surgery residency, was associated with self-assessed comfort in performing open lobectomy, suggesting that open thoracic operative competence is neither systematically taught nor reliably acquired through current training pathways [30]. This survey addresses general surgery residents rather than dedicated thoracic surgery trainees, and may therefore underestimate open thoracic exposure in programs with a formal thoracic track; it also relies on self-assessment, which is an imperfect proxy for measured competence. With these limitations acknowledged, it provides the only available programmatic data on self-assessed open competence, and its findings point to a wider systemic gap. Residents intending to pursue thoracic surgery were more comfortable with thoracotomy than their peers, but even in this self-selected group the finding suggests that open competence depends on individual motivation and opportunity rather than programmatic guarantee.

The relationship between operative volume and technical competence is a foundational principle of surgical education. Data specific to thoracic surgery confirm that, even within the minimally invasive domain, proficiency requires substantial case volume. A single-surgeon cumulative-sum (CUSUM) analysis of VATS sleeve lobectomy at a high-volume pulmonary center identified approximately 30 cases as the threshold for establishing a technical foundation and 90 cases for full proficiency [31]. A study of a junior thoracic surgeon’s early independent practice documented turning points at the 26th lobectomy and 16th segmentectomy, confirming that structured stepwise training can enable safe early-career outcomes but requires deliberate case progression [32]. If the learning curve for VATS sleeve lobectomy demands of the order of 90 cases in a dedicated high-volume center, the implication for open thoracic competence, which receives no comparable structured volume allocation in most contemporary programs, is a legitimate source of concern, even though the precise volume required to acquire and maintain open competence has not been defined.

A critical and incompletely answered question is whether competence acquired through minimally invasive surgery transfers to open surgery. VATS and open thoracic surgery share anatomical targets but differ substantially in their operative mechanics: ergonomics, spatial orientation, reliance on tactile versus visual feedback, and strategies for managing complications all differ between the two approaches. There is, at present, no established evidence demonstrating that high-volume VATS experience reliably confers readiness to manage an open thoracic field under emergency conditions; this remains an open question rather than a settled deficiency, and it is one that merits dedicated study.

Simulation offers a complementary pathway. A randomized study comparing high-fidelity 3D-printed lung models with conventional box trainers demonstrated that anatomically accurate thoracoscopic simulators produced significantly greater gains in anatomy comprehension, objective technical-skill (OSATS) scores, and operator confidence in surgical residents [33]. The conceptual framework extends to open thoracic training: simulation does not replace operative experience but may compress learning curves, permit deliberate practice of high-stakes maneuvers, and help maintain competence in procedures performed infrequently. A dedicated review of simulation training for thoracic lobectomy documents a growing landscape of simulators, from wet-laboratory and cadaveric models to virtual-reality platforms, designed to teach both VATS and open surgical skills, while noting that formal validation of most platforms remains limited and that simulators specifically targeting open pulmonary procedures are particularly scarce [34]. Of note, recent international data on minimally invasive surgery scale-up indicate that the presence of local simulation-based training facilities is independently associated with improved patient outcomes, lending empirical support to the role of simulation in the safe delivery of surgery, particularly in resource-constrained settings [35].

## 9. Preserving Open Skills: Strategies and Proposals

**Figure 2:**
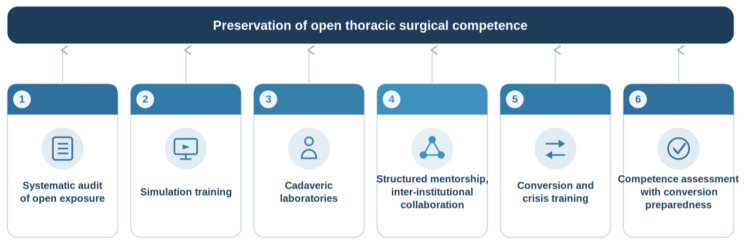
Proposed competence-preservation framework. The six elements are intended to operate together.

Against this background, and framing the issue as a foreseeable risk rather than a demonstrated decline, we propose the following responses to the potential erosion of open thoracic surgical competence. These elements are intended to function together rather than in isolation, and are summarized in Figure 2.

**Systematic audit of open surgical exposure.** Training programs and accrediting bodies may wish to consider prospectively auditing open surgical exposure, with the longer-term aim of defining evidence-based minimum thresholds. Current accreditation frameworks focus predominantly on total case volume with limited granularity regarding surgical approach. A program in which 95% of lobectomies are performed by VATS may meet aggregate volume requirements while producing trainees who have never performed a posterolateral thoracotomy independently. The introduction of specific minimum open thoracotomy case requirements as a formal component of training standards merits serious consideration by the relevant accrediting bodies.

**Simulation and cadaveric training.** Simulation-based training of the kind demonstrated for thoracoscopic procedures [33] could be systematically extended to open thoracic surgery. Thoracic-specific simulation platforms, from wet-laboratory models to cadaveric thorax preparations, may offer anatomically accurate environments for the acquisition of posterolateral thoracotomy, hilar dissection, bronchoplasty, and vascular repair, although dedicated open-pulmonary-procedure simulators remain scarce and formal validation programs are needed [34]. For complex open procedures performed in low annual volumes, simulation and cadaveric training may represent the only feasible mechanism for maintaining operative familiarity between clinical encounters.

**Structured mentorship and inter-institutional collaboration.** Visiting-fellowship models, in which trainees spend defined rotations at high-volume open surgical centers, and proctoring arrangements between experienced open thoracic surgeons and lower-volume institutions, could serve a dual function of immediate patient safety and deliberate open surgical practice for the host surgeon.

**Integrating conversion preparedness into MIS competence assessment.** Future models of competence assessment for minimally invasive thoracic surgery may need to incorporate preparedness for conversion as a core requirement. This could include documented exposure to open anatomical pulmonary resection, structured training in vascular control and crisis management, and objective assessment of the surgeon’s ability to manage an open thoracic field when required. The precise thresholds would require consensus among training bodies and professional societies, but the underlying principle is that minimally invasive competence cannot be entirely separated from the ability to perform a safe open conversion.

## 10. Conclusions

Minimally invasive surgery has profoundly reshaped thoracic surgical practice and has delivered clear benefits for patients undergoing anatomical lung resection. This progress should not be viewed as being in opposition to open surgery. Rather, the expansion of VATS and RATS has created a new educational and institutional challenge: how to preserve competence in procedures and crisis scenarios that are encountered less frequently but remain essential to comprehensive thoracic surgical care.

Open thoracic surgery is not a lost skill. The traditional conditions through which open competence was acquired (repeated exposure to thoracotomy, progressive responsibility in complex resections, and direct apprenticeship in open crisis management) are, however, becoming less common in many contemporary training environments. At the same time, open surgery remains indispensable for selected patients with complex hilar disease, bronchoplastic or angioplastic requirements, chest wall invasion, superior sulcus tumors, carinal involvement, hostile post-induction fields, contraindications to single-lung ventilation, and for all patients requiring urgent conversion from a minimally invasive approach. It also remains the predominant approach in much of the world, a reminder that this is a globally distributed competence rather than a locally restricted one.

The available evidence does not prove an irreversible decline in open surgical competence, and we do not mean to overstate it. What the evidence does identify is a credible and foreseeable risk. Reduced exposure to open procedures is documented; the transferability of minimally invasive skills to the open field is uncertain; and explicit open-competence requirements are absent from many training and competence-assessment frameworks. These observations, taken together, suggest that the issue should no longer be left to informal apprenticeship alone, without implying that competence has already been lost.

Preserving open thoracic competence should therefore be regarded not as resistance to innovation, but as a necessary condition for the safe evolution of the specialty. Systematic assessment of open operative exposure, structured simulation and cadaveric training, mentorship across high-volume centers, multidisciplinary crisis preparedness, and competence-assessment models that incorporate readiness for conversion may help ensure that future thoracic surgeons remain capable not only of performing minimally invasive procedures, but also of managing the open surgical field when patient safety requires it. Further prospective, multi-institutional study will be needed to define the operative volume required to acquire and maintain open competence, and to test whether these measures achieve their intended effect.

## Figures and Tables

**Table 1:** Clinical scenarios in which open thoracic surgery remains necessary, strongly preferred, or in which open readiness is mandatory.

Clinical Scenario	Rationale for Open Approach
Sleeve/bronchoplastic resection	Precise bronchial transection and tension-free anastomotic reconstruction.
Invasion of great vessels with resection and reconstruction	En bloc vascular resection and reconstruction; feasible only in highly selected patients at experienced centers [9].
Carinal resection and reconstruction	Apex of technical complexity; shared airway, multiple anastomotic configurations, concentration in specialized centers [10,11].
Superior sulcus (Pancoast) tumor	Multidisciplinary resection in a previously irradiated field after trimodality therapy; anatomically complex region; tissue fragility and altered planes [12].
Redo thoracic surgery/hostile pleura	Dense intrapleural adhesions (up to 76%); conversion in ~12% of thoracoscopic redo cases [13].
Post-induction (chemoimmunotherapy) fields	Fibrosis and altered hilar anatomy; conversion rate ~19%, higher than upfront surgery [14].
Intolerance of single-lung ventilation	Open thoracotomy may be the only viable approach when one-lung ventilation cannot be sustained.
Thoracic deformity	Minimally invasive access mechanically precluded or unsafe.
Emergency thoracic surgery/unplanned conversion	Rapid open vascular control required regardless of institutional MIS profile.

## Data Availability

No new data were created or analyzed in this study. Data sharing is not applicable to this article.

## Abbreviations

The following abbreviations are used in this manuscript:

CUSUM	Cumulative Sum
ERS	European Respiratory Society
ESTS	European Society of Thoracic Surgeons
MIS	Minimally Invasive Surgery
NSCLC	Non-Small Cell Lung Cancer
OSATS	Objective Structured Assessment of Technical Skills
RATS	Robotic-Assisted Thoracic Surgery
STS	Society of Thoracic Surgeons
VATS	Video-Assisted Thoracoscopic Surgery
